# The incidence risk of gynecological cancer by antipsychotic use: a meta-analysis of 50,402 patients

**DOI:** 10.1186/s12885-024-12481-6

**Published:** 2024-06-10

**Authors:** Francisco Cezar Aquino de Moraes, Renan Yuji Ura Sudo, Maria Eduarda Cavalcanti Souza, Marianne Rodrigues Fernandes, Ney Pereira Carneiro dos Santos

**Affiliations:** 1 Oncology Research Center, University Hospital João de Barros de Barreto, Federal University of Pará, Rua dos Mundurucus, n?4487, Belém, PA 66073-000 Brazil; 2 Federal University of Grande Dourados, Dourados, Mato Grosso do Sul Brazil; 3 University of Pernambuco, Recife, Pernambuco Brazil

**Keywords:** Antipsychotics, Drug Side effects, Epidemiology, Gynecological cancer, Incidence

## Abstract

**Background:**

Female gynecological cancers represent a serious public health problem, with 1,398,601 new diagnoses and 671,875 deaths per year worldwide. Antipsychotics are often used in psychiatric disorders, including schizophrenia, bipolar disorder, and major depression. It is estimated that the prescription of these drugs is linked to 1,800 deaths a year in the United States, but their association with cancer remains controversial.

**Methods:**

We searched PubMed, Scopus, and Web of Science databases for studies reporting the correlation in the incidence risk of gynecological cancer by antipsychotic use. We used DerSimonian and Laird random-effect models to compute logit transformed odds ratio (OR) for the primary binary endpoint with 95% confidence interval (CI). Heterogeneity was assessed through effect size width along with I-squared and Tau-squared statistics. Review Manager 5.4.1. was used for statistical analyses. A p-value of < 0.05 denoted statistically significant.

**Results:**

50,402 patients were included, of whom 778 (1,54%) took antipsychotic medication for at least 1 year. 1,086 (2,15%) with ovarian cancer and 49,316 (97,85%) with endometrial cancer. Antipsychotic use (OR 1.50; 1.06 to 2.13 95% CI; p-value 0.02), hypertension (OR 1.50; 95% CI 1.06 to 2.13; p-value < 0.01), nulliparity (OR 1.98; 95% CI 1.53 to 2.57; p-value < 0.01) and multiparity (OR 0.53; 95% CI 0.41 to 0.69; p-value < 0.01) showed significantly different distributions between groups of cancer and cancer-free patients. The primary endpoint of incidence risk of gynecological cancer by antipsychotic therapy showed a statistically significant difference (OR 1.67; 95% CI 1.02 to 2.73; p-value < 0.05) against the use of antipsychotic drugs.

**Conclusions:**

Our meta-analysis showed that the use of antipsychotic drugs increases the risk of gynecological cancers, particularly endometrial cancer. This result should be weighed against the potential effects of treatment for a balanced prescribing decision.

**Supplementary Information:**

The online version contains supplementary material available at 10.1186/s12885-024-12481-6.

## Introduction

Cancer remains a formidable public health challenge, posing a significant burden on individuals, healthcare systems, and society as a whole. Despite remarkable progress in cancer research and treatment, the disease continues to inflict a significant toll, with demographic and epidemiological shifts leading to a rise in cancer incidence, with nearly two million new cases diagnosed worldwide in 2020 alone [1–3]. Gynecological cancers comprise a group of neoplasms that arise in the reproductive or genital organs of women, predominantly cervical cancer (CC), endometrial cancer (EC), and ovarian cancer (OC) [4]. They represent a worldwide public health problem, and according to Global Cancer Incidence, Mortality, and Prevalence (GLOBOCAN), there were 1,398,601 diagnosed cases of gynecological cancer and 671,875 associated deaths worldwide in 2020 [5]. Currently, the standard of care for these tumors includes surgical resection, chemotherapy, and immunotherapy with immune checkpoint inhibitors [5–7].

Interestingly, people living with mental illness, with or without the use of APDs, have a lower risk of developing cancer [8–10]. Currently, some hypotheses may explain the low frequency of cancer in this population. First, genetic factors may play a protective role in mental illnesses, such as schizophrenia, a disease that has previously been associated with high p53 expression as being involved in its etiology [11, 12]. The p53 gene is a tumor suppressor and a candidate for lowering the frequency of lung cancer and other cancers in patients with schizophrenia [13]. Second, the long-term use of APDs is associated with a decrease in life expectancy among users compared with that in the general population [14, 15]. Thus, as cancer is a disease that is often related to aging, this could reflect an artificial reduction in the incidence of cancer among patients who use APDs [11].

Antipsychotic drugs (APDs) are a class of medication commonly used in psychiatric disorders, including schizophrenia, bipolar disorder, major depression, and personality disorders [16]. A study conducted by the United Kingdom(UK) Department of Health showed that of 180,000 prescriptions analyzed, at least 140,000 were considered inappropriate, of which APDs were observed to be extremely harmful to users [17]. It is estimated that there are 1,800 deaths a year due to the use of APDs in the UK alone. In the United States of America (USA), 75,000 patients aged ≥ 65 years from the Centers for Medicare and Medicaid Services (CMS) showed increased mortality with all APDs except quetiapine [15, 18]. Therefore, in 2005, after observing a 60–70% increase in the risk of death associated with the use of APDs in patients with dementia, the Federal Food and Drug Administration (FDA) required that warnings be added to the labels of these drugs [19].

We conducted a systematic review and meta-analysis to assess whether exposure to antipsychotics is associated with the risk of developing gynecological cancers.

## Methods

### Protocol and registration

This systematic review followed the Cochrane’s Collaboration and the Preferred Reporting Items for Systematic Reviews and Meta-Analysis (PRISMA) guidelines [20]. The protocol was prospectively registered in the International Prospective Register of Systematic Reviews (PROSPERO) with registration number CRD42023486704.

The studies were selected on the basis of the PECO question, including human studies (P-people) in which antipsychotics were used (E-exposure) or not (C-comparison) to observe an association between these medications and gynecological cancers (O-result). Thus, we sought to answer the following question: Is there an association between the incidence risk of gynecological cancer and the use of antipsychotic medication?

### Eligibility criteria

Studies that met the following eligibility criteria were included: (1) case-control or observational studies; (2) report correlation in the incidence risk of gynecological cancer by antipsychotics use; (3) adult patients taking antipsychotics for at least 1 year; (4) no previous cancer or anti-cancer therapy. We excluded studies (1) with overlapping populations; (2) without outcomes of interest; (3) with unpublished results, (4) grey literature.

### Search strategy and data extraction

Pubmed, Web of Science, and Scopus were systematically searched through October 2023. The complete search strategy used in this search were: “Psychotropic drugs”, “Hallucinogens”, “Antipsychotic Agents”, ”Phenothiazines”, “Haloperidol”, “Lithium”, “Loxapine”, “Molindone”, “Pimozide”, “Aripiprazole”, “Clozapine”, “Lumateperone”, “Lurasidone”, “Olanzapine”, “Paliperidone”, “Quetiapine”, “Risperidone”, “Ziprasidone”, “Endometrial Neoplasms”, “Uterine Cervical Neoplasms”, “Female Genital Neoplasms”, “Ovarian Neoplasms”, “Fallopian Tube Neoplasms”, “Uterine Neoplasms”, “Vagina Neoplasms”, “Vulva Neoplasms”, “Ovarian Epithelial Carcinoma”, “Gonadal Tissue Neoplasms”. The search strategy with the MeSH terms and boolean operators is more detailed in Table S2, Supplementary Material.

Aiming for the inclusion of additional studies, the references of the included articles and systematic reviews of the literature were evaluated and an alert was established for notifications in each database, in case a study corresponding to the consultation carried out was eventually published. Those found in the databases and the references of the articles were incorporated into the reference management software Rayyan®. Duplicate articles were removed, using both automated and manual methods. Subsequently, three reviewers (F.C.A.M., M.E.C.S., and R.Y.U.S.) independently analyzed the titles and abstracts of the identified articles. Disagreements were resolved by consensus between the three authors and the senior author (F.C.A.M, M.E.C.S., R.Y.U.S., and N.P.C.S).

The following baseline characteristics were extracted: (1) study name, year and design; (2) country; (3) number of patients allocated for each arm; (4) cancer type; (5) age; (6) regimen details in experimental and control arm; and (7) main patient characteristics, namely diabetes, hypertension, parit and smoking. Three authors (F.C.A.M, M.E.C.S., and R.Y.U.S.) independently collected pre-specified baseline characteristics and outcome data. Disparities were resolved by consensus.

### Endpoints

The primary outcome of interest for a pooled analysis was the incidence of gynecological cancer. In our study, we defined gynecological cancer as any malignant neoplasm that originated in the endometrium, cervix, vagina, vulva, or ovary.

Psychotropic medication was defined as first-generation typical antipsychotics, including Phenothiazines, Chlorpromazine, Thioridazine, Trifluoperazine, Fluphenazine, Perphenazine, Prochlorperazine, Haloperidol, Loxapine, Molindone, Pimozide, Lithium, and second-generation atypical antipsychotics, comprising Clozapine, Olanzapine, Risperidone, Quetiapine, Ziprasidone, Paliperidone, Aripiprazole, Asenapine, Iloperidone, Lurasidone, Brexpiprazole, Cariprazine, Pimavanserin, Lumateperone [21].

### Risk of bias assessment

The quality assessment of individual observational studies was carried out using the Newcastle-Ottawa Scale for non-randomised studies [22]. Two authors (F.C.A.M., and R.Y.U.S.) independently conducted the assessment, and disagreements were resolved by consensus or adjudication with a third author (M.E.C.S). Each trial was evaluated in three different domains, namely: selection of exposed cohorts, external controls, exposures and outcomes of interest; comparability of main and additional factors; outcome assessment, follow-up time, and adequacy of follow-up time. To examine publication bias, contour-enhanced funnel plots were visually inspected and assessed by Egger’s regression asymmetry and Begg’s rank correlation test [23].

### Statistical analysis

Pertinent baseline characteristics of the included sample were pooled to test the probability of their effects on the outcome. Logit transformation was performed on the reported odds ratios (OR) to compute the binary outcome of interest with a 95% confidence interval (CI). The width of effect sizes along with I^2^ and Tau^2^ statistics were used to assess heterogeneity [24]. We used DerSimonian and Laird random-effect models for the primary endpoint [25]. Publication bias was explored using Begg’s precision of effect sizes and Egger’s linear regression test [26]. Statistical analyses were performed using Review Manager 5.4.1 and R software, version 4.2.1.

## Results

### Study selection and baseline characteristics

**Fig. 1:**
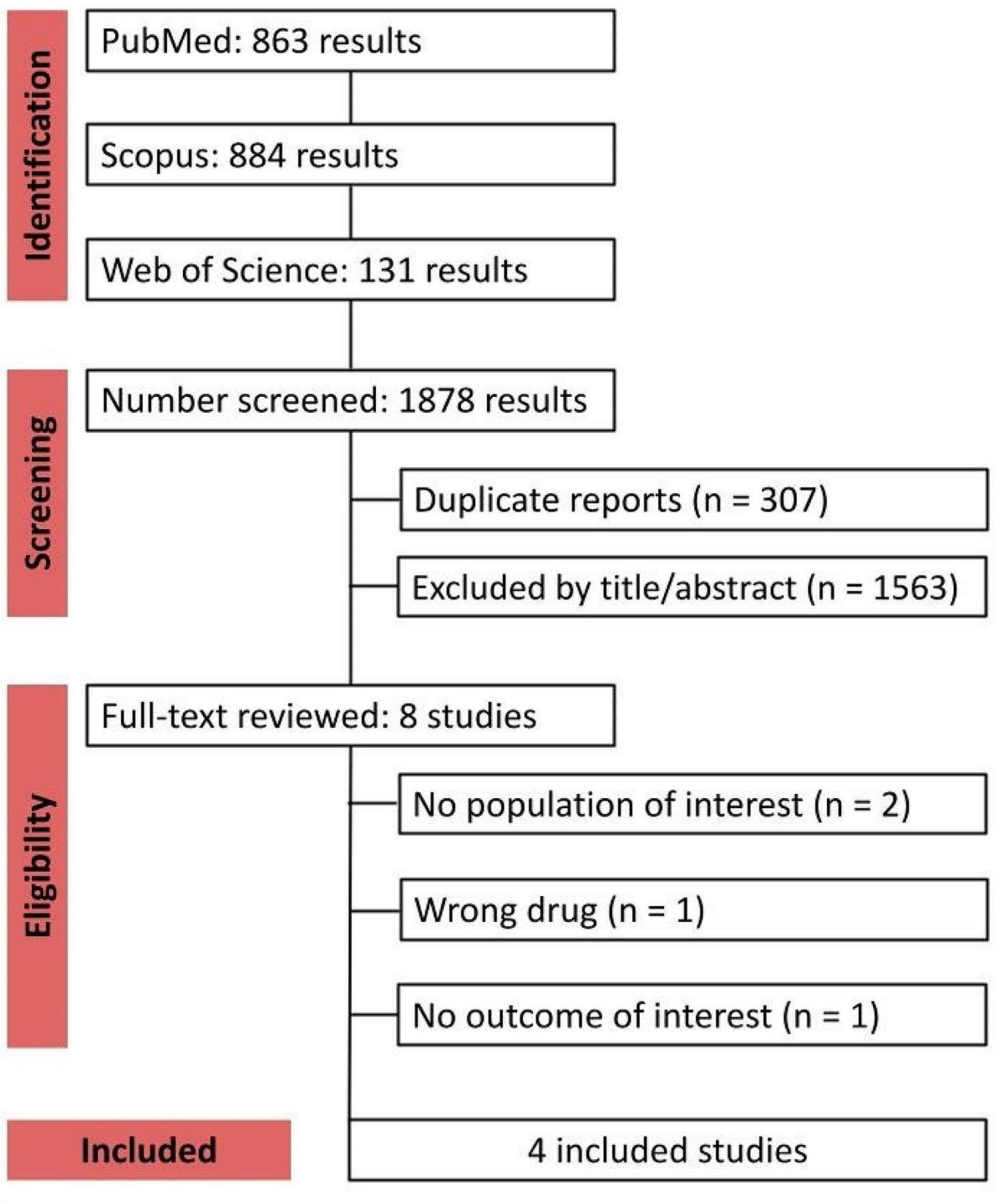
PRISMA flow diagram

As described in the PRISMA flow diagram (Fig. 1), a total of 1,878 references were retrieved in our systematic search. After removing duplicates and screening titles or abstracts, 8 full-text manuscripts were eligible and thoroughly reviewed for inclusion and exclusion criteria. Ultimately, 4 observational studies satisfied the eligibility criteria and formed the scope of the analysis [27–30]. References for the excluded studies after full-text review are available in the Supplemental Appendix.

A total of 50,402 women with gynecological cancer were analyzed for the association of cancer with antipsychotics. Most of the patients were diagnosed with endometrial cancer (*n* = 49,316, 97,85%); 1,086 (2,15%) with ovarian cancer. The baseline characteristics of the included studies are summarized in Table 1. According to the available data, the number of women who have reported as being nulliparous is 332, and 918 were multiparous. 1,243 patients had a history of smoking. Regarding metabolic characteristics, 16,022 (31,79%) patients had Hypertension, and 7,800 (15,48%) had Diabetes Mellitus. The majority of the patients were treated with prolactin-elevating antipsychotics. Most of the patients were aged above 65 years, with 283 women under the age of 40.

**Table 1:** Design and Characterístics of Studies Included in the Meta-analysis

Author§	Country	Design	Cancer type	No. of patients		Age§		Antipsychotic drugs
Chen [28], 2022	Taiwan	Case-control	Endometrial cancer	9502	37,908	≥ 65: 1,421 (15.0)	≥ 65: 5,645 (14.9)	Quetiapine; Haloperidol; Risperidone; Olanzapine; Amisulpride; Clozapine; Aripiprazole
Harlow [27], 1998	United States of America	Cohort study	Ovarian cancer	563	523	< 40: 107 (19)	< 40: 126 (24)	Phenothiazines, lithium, haloperidol triavil
Klil-Drori [29], 2017	United Kingdom	Case-control	Endometrial cancer	139	1603	62.2 (14.3)	62.2 (14.9)	Benperidol Aripiprazole Chlorpromazine Asenapine Droperidol Clozapine Flupentixol Olanzapine Fluphenazine Quetiapine Fluspirilene Sertindole Haloperidol Levomepromazine Loxapine Oxypertine Pericyazine Perphenazine Pimozide Pipotiazine Promazine Sulpiride Thioridazine Trifluoperazine Trifluperidol Zuclopenthixol Amisulpride Paliperidone Risperidone Zotepine
Yamazawa [30], 2003	Japan	Case-control	Endometrial cancer	41	123	< 40: 13 (32)	< 40: 37 (30)	Sulpiride; Levomepromazine; Sultopride; Chlorpromazine; Haloperidol; Risperidone; Chlorpromazine; Pimozide; Clocapramine

No: number of patients; §: years

### Estimation of group-assignment imbalance of main baseline characteristics

The assessment of p-value for demographic data is outlined in Table 2. Antipsychotic use (p-value 0.02), hypertension (p-value < 0.01), nulliparity (p-value < 0.01), and multiparity (p-value < 0.01) showed significant differences in the distribution between groups of cancer and cancer-free patients. Forest plots for each characteristic are displayed in the Supplement material.

**Table 2:** Pooled analysis of main baseline characteristics

Characteristics	*N* of studies	*N* of Patients	OR	95% CI	*p*-Value	Heterogeneity				Subgroup difference
						Tau²	df	*p*-Value	I²	
Antipsychotic Use	4	50,402	1.50	1.06 to 2.13	0.02*	0.07	3	0.05	61%	NA
Diabetes	3	49,316	1.89	0.97 to 3.69	0.06	0.25	2	0.007	80%	
Hypertension	3	16,067	1.42	1.36 to 1.49	< 0.01*	0.00	2	0.54	0%	
Parity	2	2,500	1.11	0.47 to 2.63	< 0.01*	0.69	3	< 0.01	94%	
*0	2	1,250	1.98	1.53 to 2.57	< 0.01*	0.00	1	0.52	0%	p-value < 0.00001*
*1≥	2	1,250	0.53	0.41 to 0.69	< 0.01*	0	1	0.63	0%	
Smoking	2	5,656	1.00	0.87 to 1.15	0.99	0.00	3	0.51	0%	NA
*Ever	2	2,828	1.08	0.88 to 1.32	0.41	0.00	1	0.41	0%	*p*-value 0.31
*Never	2	2,828	0.93	0.76 to 1.13	0.45	0.00	1	0.45	0%	

CI: confidence interval; N: number of patients; NA: not applicable; OR: odds ratio; * significant p-value

### Incidence of gynecological cancer

**Fig. 2:**
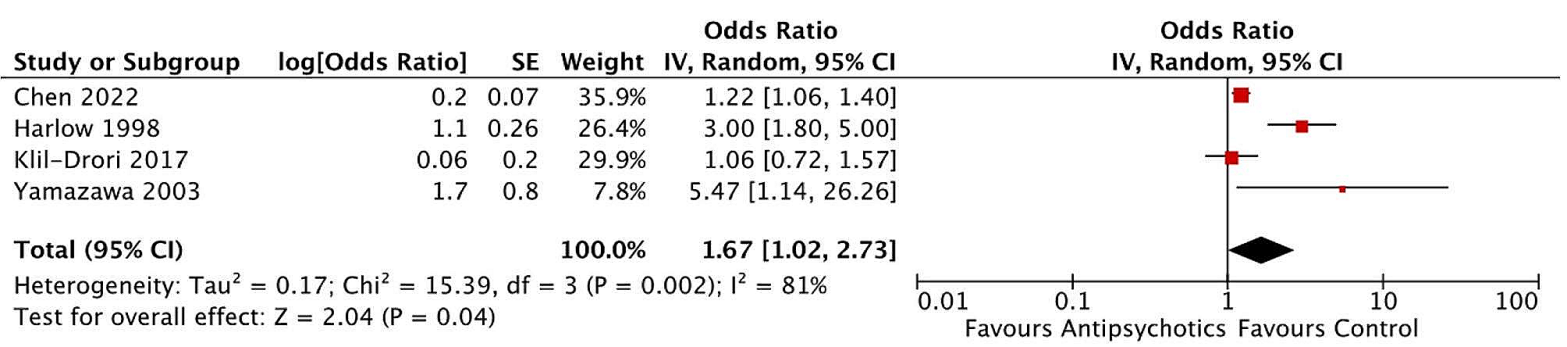
Antipsychotic drug exposure increases the likelihood of gynecological cancer by a mean 1.67-point

Four observational studies of 50,402 patients reported the odds ratio for gynecological cancer in individuals undergoing antipsychotic therapy. In the pooled analysis, a statistically significant 1.67-point (67%) increased logit-transformed odds was observed in the antipsychotic group (OR 1.67; 95% CI 1.02 to 2.73; *p* < 0.05; Fig. 2). A Z-value for a test of the null hypothesis is 2.04 with a corresponding p-value of 0.04. Between-study variation of observed effects is estimated by an I-squared value of 81% along with an absolute true effect size variance estimated by a Tau-squared value of 0.17.

### Estimation of publication bias

**Fig. 3:**
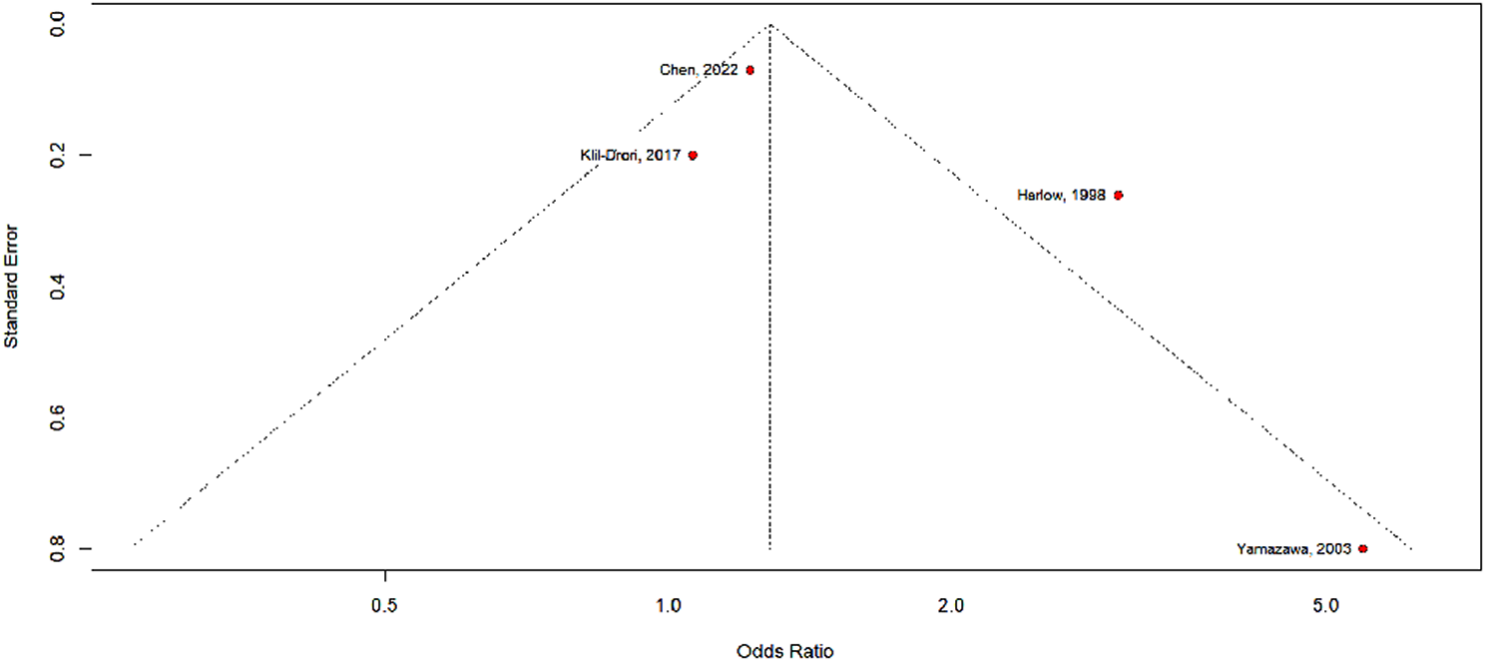
Funnel plot comparison of studies’ effect size to index of precision for analysis of publication bias

Figure 3 shows the funnel plot of the included articles for publication bias assessment. The X-axis corresponds to the odds ratio, while the Y-axis represents the standard error. The dashed lines indicate two standard errors on either side of the mean effect. Each circle is representative of one study. Test for asymmetry was statistically significant by Begg’s rank correlation between precision and effect size, and Egger’s regression test. If the small-study effect from Yamazawa et al. was due to publication bias, then the true effect size would be smaller than our estimate.

### Quality assessment

Table 3 outlines a detailed evaluation of each study included in this meta-analysis performed by two independently paired reviewers (F.C. and R.S.). Overall, 3 of 4 studies were deemed at good quality. The study by Harlow et al. was considered to be of poor quality because not fulfill the minimum criteria for outcome follow-up domains.

**Table 3:** The Newcastle-Ottawa Scale (NOS) for quality evaluation of the included non-randomized studies

Study		Selection				Comparability		Outcome			Total (9/9)
		Representative of the exposed cohort	Selection of external control	Ascertainment of exposure	Outcome of interest not present at the start of the study	Main factor	Additional factor	Assessment of outcomes	Sufficient follow-up time	Adequacy of follow-up	
Chen 2022		☆	☆	☆	☆	☆	☆	☆	☆	☆	9
Harlow 1998		☆	☆	☆	☆	☆	☆	☆	0	0	7
Klil-Drori 2017		0	☆	☆	☆	☆	☆	☆	☆	☆	8
Yamazawa 2003		0	☆	☆	☆	☆	☆	☆	☆	☆	8

*Abbreviations* *Study design: Prospective (+), Retrospective (−); single centre (−), multicentre (+), Maximum quality score = 9; 0–7 points were considered lower quality, and 8–9 points were considered as higher quality

## Discussion

In this systematic review and meta-analysis including 50,402 patients, we assessed the correlation between the incidence risk of gynecological cancer and the use of antipsychotic medications. The primary finding indicates that individuals undergoing antipsychotic drug regimens face a 1.67-fold likelihood of developing gynecological cancer compared to treatment-free patients. To the best of our knowledge, this is the first comprehensive review and pooled analysis of the existing body of evidence of an association between antipsychotic drugs and gynecological cancers.

Clinical practice guidelines endorse a minimum effective antipsychotic dose for the treatment of schizophrenia and bipolar disorder [31]. However, mounting evidence suggests a trend in off-label prescriptions that lack supporting evidence for effectiveness and safety [32, 33], leading to concerns of potential adverse events, especially for patients in need of a longer period of treatment. Although less explored than commonly reported adverse events, such as postural hypotension, dizziness, and sedation, possible long-term carcinogenesis associated with antipsychotic drugs has been extensively explored [34–36]. However, it warrants mention that confoundings may play a role as no randomized study up to this date has been performed. Notably, schizophrenia and bipolar disorder have been associated with increased risk of certain cancer types in a pooled analysis after adjustment for confounders [37].

Prevailing consensus suggests that the onset of most endometrial carcinomas is due to estrogen-driven endometrial proliferation [38]. Similarly, extensive research has been conducted on the association between estrogen and ovarian cancer. Notably, an individual patient data meta-analysis of 52 epidemiologic studies revealed an increased risk of ovarian cancer in a 5-year exposure to estrogen replacement therapy [39]. Johansson et al. conducted a Mendelian randomization study of 66450 patients, detecting a significant effect of estradiol on the incidence of ovarian cancer [40]. LaBella et al. identified structural similarities between certain antipsychotics, antihistamines, and tamoxifen. These drugs were also found to induce cytochrome P450 activity, which has been correlated with tumorigenic properties. While the association between tamoxifen and endometrial cancer risk is well-established, limited evidence currently exists to suggest a direct adverse effect of antipsychotics on the endometrium [30, 41]. Additionally, elevated levels of serum prolactin in ovarian and endometrial cancers have been previously reported [42, 43], some extending these findings to a potential independent risk factor for gynecological cancer [44]. However, the precise underlying mechanistic pathways remain undefined. Nonetheless, it is reasonable to anticipate a direct effect of prolactin on the endometrium and ovarium since the abnormal proliferation of these tissues is observed in hyperprolactinemia women in the presence of estradiol [42].

Antipsychotic medications are prominently associated with hyperprolactinemia in more than two-thirds of individuals undergoing treatment with these agents [45]. The primary cause may stem from the antagonistic action on dopamine D2 receptors (D2R) more frequently associated with high-potency typical antipsychotics, hypothesized to be due to a longer binding duration between the drug and D2R [46]. Although certain atypical AP also exhibit a notable potential for elevated prolactin levels [45], the majority presents a higher risk of inducing metabolic syndrome [47]. However, robust evidence to substantiate these distinctions is still lacking. Klil-Drori et al. [29] a case-control study encompassing more than 65,000 patients over 24 years, found no odds disparity between prolactin-elevating and prolactin-sparing AP. The observed 1-point odds ratio indicates that prolactin may not function as an independent risk factor but rather as a mediator of the aforementioned estrogen pathway. Similarly, Bilici et al’s [48] investigation also found no significant correlation. Studies have shown that the blockade of the pulsatile secretion of follicle-stimulating and luteinizing hormones at the hypothalamus-pituitary axis, caused by induced hyperprolactinemia, is correlated to hormonal imbalance and metabolic alterations like obesity and insulin resistance. These independent variables have also been associated with endometrial, ovarian and breast cancer [30, 49, 50]. Although prolactin itself doesn’t promote a mitogenic effect [51], hyperprolactinemia is often the cause of secondary amenorrhea, which leads to prolonged unopposed estrogen exposure that increases the risk of gynecological malignancies [28, 30]. Moreover, hyperprolactinemia alterations also affect the immunological systems, leading to inflammation-like effects and decreased immunity, such as abnormal autoantibody expression, a lower natural killer (NK) cell number, and dysregulated T cell function in comparison with women with normal prolactin levels [28, 52–54]. The association of metabolic alterations and hyperprolactinemia resulting from antipsychotics requires further detailed analysis as the risk factors involved in cell carcinogenesis may be explained by more than one physiological mechanism [55].

Conversely, Chen et al [28] in a population-based study involving 47,414 patients, reported a stronger association between haloperidol, a typical antipsychotic, and the odds of developing endometrial neoplasms, while other antipsychotics did not reach statistical significance. Zhuang et al’s [56] in a systematic review and meta-analysis comprising 160,727 patients found no association between the use of antidepressant therapies and an increased risk of breast and gynecological cancers. Nevertheless, Yamazawa et al [30] identified a considerable association between a 5-point adjusted odds ratio against antipsychotic use concerning endometrial cancer development. These nuanced findings emphasize the complex interplay between AP medications, hyperprolactinemia, and gynecological outcomes, calling for further exploration to elucidate the underlying mechanisms and ascertain the clinical implications of these associations.

In light of this, given the adverse events related to inappropriate prescription of antipsychotic drugs [57, 58], the increased odds of carcinogenesis reported in previous studies [59], and the significant association to gynecological cancer found in this study, APDs should be individualized to offer lower safety risks. Also, patients should be periodically reassessed to ascertain the continued necessity of the prescribed regimen, thereby fostering a paradigm of care that is adaptive and evidence-based.

Our study has some limitations. Most importantly, the modest number of included studies limits the robustness of our findings, as evidenced by analysis of the funnel plot that indicates a requisite for additional studies. Moreover, the lack of data on stratified outcomes by drugs and dosages hampers a more detailed examination and a subgroup analysis of cancer linear dose–response association between the AP use and incidence risk of the gynecological cancer. Furthermore, the absence of data did not allow for the reporting of important details, including the patients’ diverse characteristics, such as post-menopausal, menopausal, and pre-menopausal stages, clinical history, lifestyle factors, and socioeconomic background. Lastly, a high between-study variation across the spectrum of results may bias our pooled analysis despite the narrow-ranging effect size, minimized by the use of the random effect in the statistical analysis.

Despite its limitations, our study has many strengths. Firstly, the population recruited for the studies encompassed different baseline population characteristics and a wide range of ages. This supports the potential of the patient-specific factors that may interact with antipsychotic use and influence the risk of gynecological cancers. In addition, the number of people recruited comprises a large sample per the reality of available evidence to date. The findings of our study provide insights into the potential carcinogenic properties of AP, suggesting the need for further investigation into its role in cancer development. The insights presented in this article could prove valuable in guiding future screening and monitoring protocols.

## Conclusions

The results of this Systematic Review and Meta-analysis involving the largest sample size to date and mostly included comprehensive observational studies provide robust evidence that the use of antipsychotics is associated with an increased risk of gynecological cancers, particularly endometrial and ovarian cancer. These findings support the idea that antipsychotics should be prescribed with caution. Future studies analyzing the association of AP with gynecological cancers with longer follow-up periods and drug-stratified analysis, specifying dose or duration, are needed to accurately assess and clarify this potential risk association and its biological mechanism.

### Electronic supplementary material

Below is the link to the electronic supplementary material.

Supplementary Material 1

## Data Availability

All data generated and/or analysed during this study are included in this published article [and its supplementary information files].

## Abbreviations

APD(s): Antipsychotic drug(s)
CC: Cervical cancer
Cls: Confidence intervals
CMS: Centers for medicare and medicaid services
EC: Endometrial cancer
FDA: Federal food and drug administration
GLOBOCAN: Global cancer incidence, mortality, and prevalence
OC: Ovarian cancer
OR: Odds ratio
PRISMA: Preferred reporting items for systematic reviews and meta-analysis
PROSPERO: International prospective register of systematic reviews
UK: United Kingdom
USA: United States of America
