# Supplementary material for: The incidence risk of gynecological cancer by antipsychotic use: a meta-analysis of 50,402 patients
Source: BMC Cancer. 2024 Jun 10;24:712. doi: 10.1186/s12885-024-12481-6 (PMC11163728; doi:10.1186/s12885-024-12481-6)
Supplement: Supplementary file 1 — Supplementary Material 1 [file 12885_2024_12481_MOESM1_ESM.docx]

**SUPPLEMENTARY MATERIAL**

Full Search Strategy

(“Psychotropic medication” OR "atypical antipsychotic drug" OR "atypical antipsychotic medication" OR "atypical antipsychotic medications" OR "typical antipsychotic medication" OR "typical antipsychotic medications" OR "atypical antipsychotic agent" OR "atypical antipsychotic agents" OR "typical antipsychotic agent" OR "typical antipsychotic agents" OR "atypical antipsychotic drugs" OR "typical antipsychotic drug" OR "typical antipsychotic drugs" OR “Psychotropic medications” OR “Psychotropic Drug” OR “Psychoactive Agent” OR “Psychoactive Drug” OR Psychopharmaceuticals OR “Psychoactive Agents” OR “Psychoactive Drugs” OR “Psychotropic Drugs” OR Psychopharmaceutical OR antipsychotics OR antipsychotic OR Thymoanaleptics OR Thymoanaleptic OR Thymoleptic OR Thymoleptics OR “Psychedelic Agents” OR “Psychedelic Agent” OR “Psychotomimetic Agents” OR “Psychotomimetic Agent” OR “Hallucinogenic Agents” OR “Hallucinogenic Agent” OR “Hallucinogenic Drugs” OR “Hallucinogenic Drug” OR Hallucinogen OR Hallucinogens OR  “Antipsychotic Drug” OR “Antipsychotic Drugs” OR “Antipsychotic Agents” OR “Antipsychotic Agent” OR “Antipsychotic Medication” OR “Antipsychotic Medications” OR “Neuroleptic Agents” OR “Neuroleptic Agent” OR “Neuroleptic Drugs” OR “Neuroleptic Drug” OR Neuroleptics OR Neuroleptic OR Phenothiazines OR Chlorpromazine OR Fluphenazine OR Perphenazine OR Prochlorperazine OR Thioridazine OR Trifluoperazine OR Haloperidol OR Lithium OR  Loxapine OR Molindone OR Pimozide OR Aripiprazole OR Asenapine OR Brexpiprazole OR Cariprazine OR Clozapine OR Iloperidone OR Lumateperone OR Lurasidone OR Olanzapine OR Paliperidone OR Pimavanserin OR Quetiapine OR Risperidone OR Ziprasidone) AND ("Endometrial Neoplasm" OR "Endometrial Neoplasms" OR "endometrial Carcinoma" OR "Endometrial Carcinomas" OR "Endometrial cancer" OR "Endometrial Cancers" OR "Endometrium cancer" OR "Endometrium Neoplasms" OR “Endometrium Neoplasm" OR "Endometrium Cancers" OR "Cancer of the Endometrium" OR "Carcinoma of Endometrium" OR "Endometrium Carcinoma" OR "Endometrium Carcinomas" OR "Cancer of Endometrium" OR "endometrial carcinosarcoma” OR “Endometrioid Carcinoma” OR “Endometrioid Carcinomas” OR “Endometrioid Adenocarcinoma” OR “Endometrioid Adenocarcinomas” OR “Endometrial Stromal Tumor” OR “Endometrial Stromal Tumors” OR “Endometrial Stromal Low-grade sarcoma” OR “Endolymphatic Stromal Myosis” OR “Endolymphatic Stromal Myoses” OR ”Endometrial Stromal Sarcoma” OR “Endometrial Stromal Sarcomas” OR “Uterine Cervical Neoplasm” OR “Uterine Cervical Neoplasms” OR “Cervical Neoplasms” OR “Cervical Neoplasm” OR “Cervix Neoplasm” OR “Cervix Neoplasms” OR “Cancer of the Uterine Cervix” OR “Cancers of the Uterine Cervix” OR “Cancer of the Cervix” OR “Cancers of the Cervix” OR “Cervical Cancer” OR “Cervical Cancers” OR “Uterine Cervical Cancer” OR “Uterine Cervical Cancers” OR “Cancer of Cervix” OR “Cancers of Cervix” OR “Cervix Cancer” OR “Cervix Cancers” OR “Gynecologic Neoplasms” OR “Gynecologic Neoplasm” OR “Female Genital Neoplasms” OR “Female Genital Neoplasm” OR “Ovarian Neoplasm” OR “Ovarian Neoplasms” OR “Ovary Neoplasm” OR “Ovary Neoplasms” OR “Ovary Cancers” OR “Ovary Cancer” OR “Ovarian Cancers” OR “Ovarian Cancer” OR “Cancer of Ovary” OR “Cancers of Ovary” OR “Cancer of the Ovary” OR “Cancers of the Ovary” OR “Fallopian Tube Neoplasms” OR “Fallopian Tube Neoplasm” OR “Fallopian Tube Cancers” OR “Fallopian Tube Cancer” OR “Cancer of the Fallopian Tube” OR “Cancers of the Fallopian Tube” OR “Uterine Neoplasms” OR “Uterine Neoplasm” OR “Uterus Neoplasms” OR “Uterus Neoplasm” OR “Cancers of Uterus” OR “Cancer of Uterus” OR “Uterus Cancer” OR “Uterus Cancers” OR “Cancer of the Uterus” OR “Cancers of the Uterus” OR “Vagina Neoplasm” OR “Vagina Neoplasms” OR “Vagina Neoplasms” OR “Vagina Neoplasm” OR “Vaginal Cancer” OR “Vaginal Cancers” OR “Cancer of the Vagina” OR “Cancers of the Vagina” OR “Cancer of Vagina” OR “Cancers of Vagina” OR “Vagina Cancers” OR “Vagina Cancer” OR “Vulva Neoplasm” OR “Vulva Neoplasms” OR “Vulva Neoplasms” OR “Vulva Neoplasm” OR “Cancer of Vulva” OR “Cancers of Vulva” OR “Vulva Cancer” OR “Vulva Cancers” OR “Cancer of the Vulva” OR “Cancers of the Vulva” OR “Vulvar Cancer” OR “Vulvar Cancers” OR “Ovarian Epithelial Carcinomas” OR “Ovarian Epithelial Carcinoma” OR “Epithelial Ovarian Cancers” OR “Epithelial Ovarian Cancer” OR “Ovarian Epithelial Cancers” OR “Ovarian Epithelial Cancer” OR “Ovarian Epithelial Carcinomas” OR “Ovarian Epithelial Carcinoma” OR “Epithelial Ovarian Carcinomas” OR “Epithelial Ovarian Carcinomas” OR “Gonadal Tissue Neoplasms” OR “Gonadal Tissue Neoplasm”)

Forest plots for group-assigment imbalance of main baseline characteristics

Figure. 1S. Antipsychotic use reached statistical significance for between-groups differences.


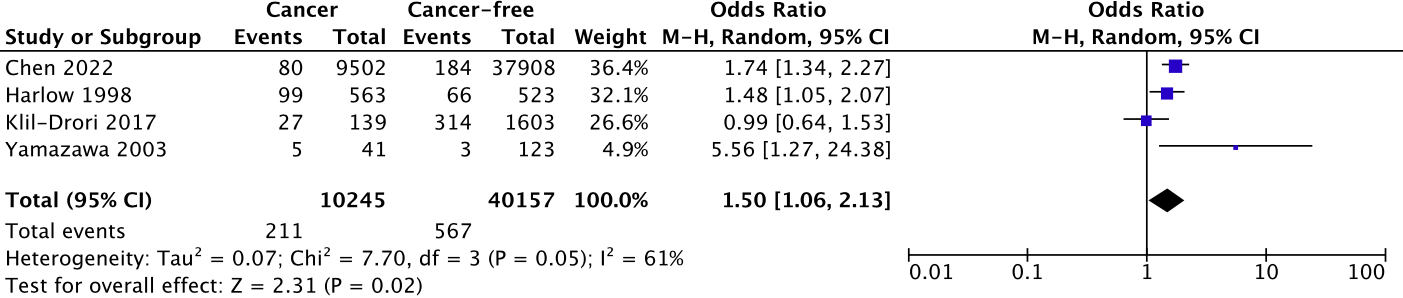


Figure. 2S. Diabetes did not reach statistical significance for between-groups differences.


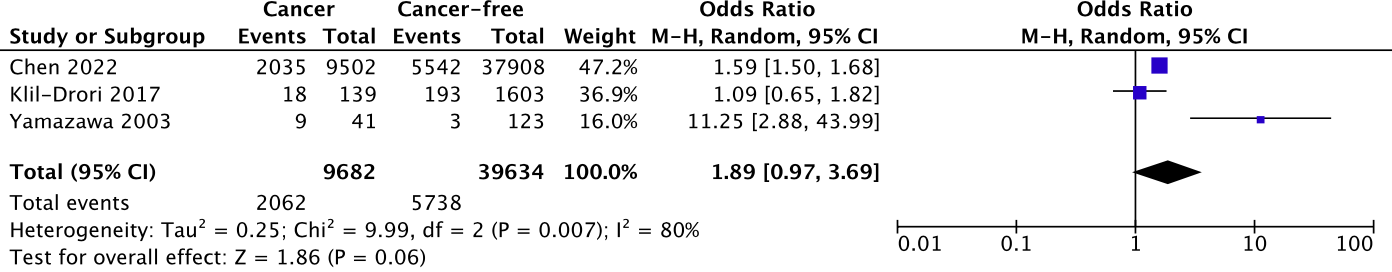


Figure. 3S. Hypertension reached statistical significance for between-groups differences.


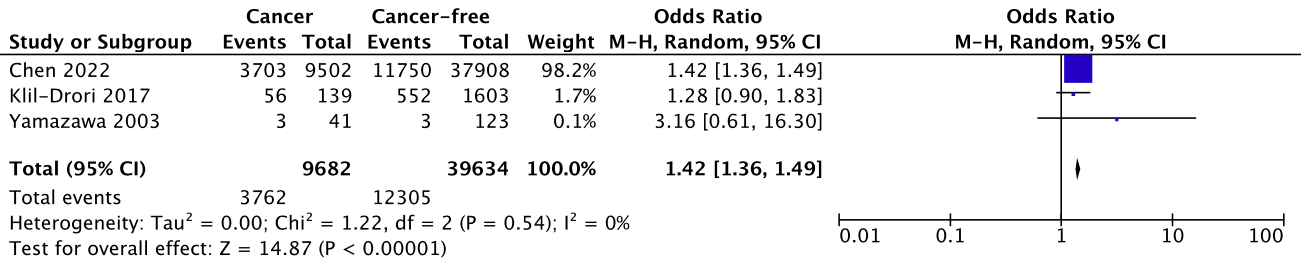


Figure. 4S. Parity did not reach statistical significance for between-groups differences. Multiparity reached statistical significance in favor of cancer group, while nulliparity was more prevalent in cancer-free group.


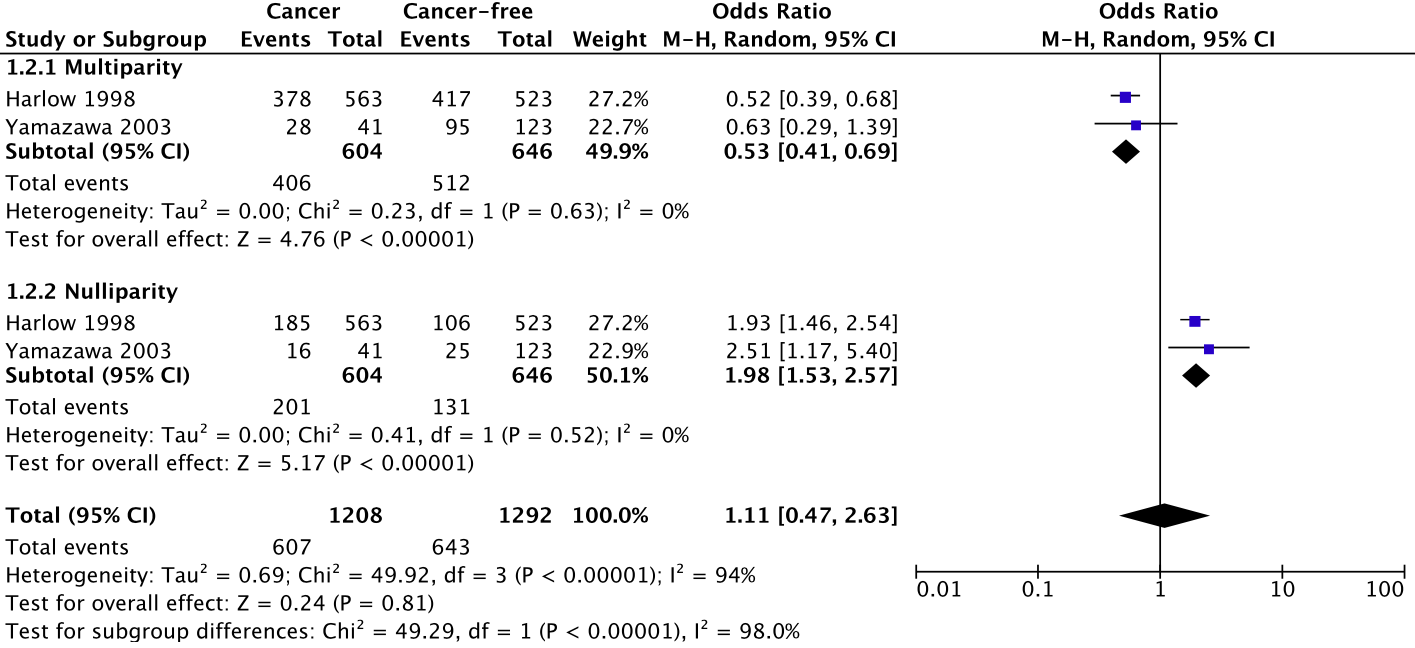


Figure. 5S. Smoking did not reach statistical significance for between-groups differences.


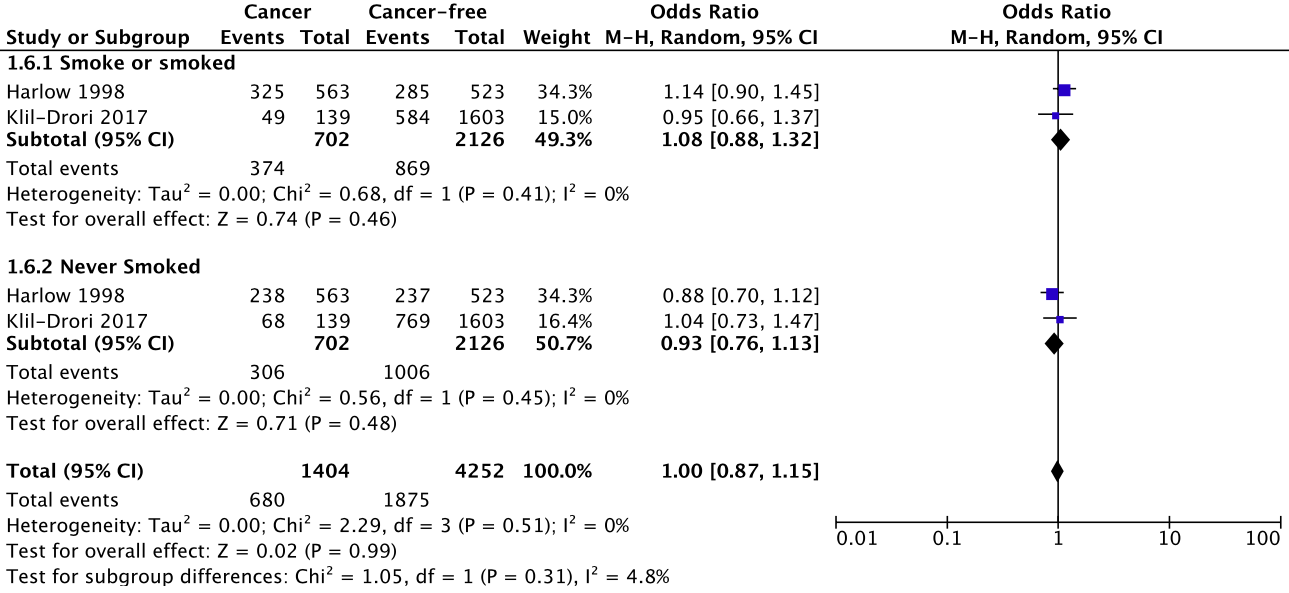


Reasons for exclusion after full-text review

As shown in Figure 1, we selected a total of 8 full-text review. Two of them were excluded for addressing different population of interest – no adult patients taking antipsychotics for at least 1 year with gynecological cancer (1,2). One study was excluded for having a different intervention arm, not using antipsychotics (3). Finally, one study was excluded for not having any outcome of interest (4). The remaining 4 studies were deemed eligible according to the prespecified criteria cited in the methods section.

1. Mortensen PB (1987) Neuroleptic treatment and other factors modifying cancer risk in schizophrenic patients. Acta Psychiatrica Scandinavica 75, 585–590.

2. Taipale H, Solmi M, Lähteenvuo M, Tanskanen A, Correll CU and Tiihonen J (2021) Antipsychotic use and risk of breast cancer in women with schizophrenia: a nationwide nested case-control study in Finland. The Lancet Psychiatry 8, 883–891.

3. Moorman PG, Berchuck A, Calingaert B, Halabi S, Schildkraut JM. Antidepressant medication use for and risk of ovarian cancer. Ob Gynecol (2005) 105(4):725-30.

4. Coogan PF, Rosenberg L, Palmer JR, Strom BL, Stolley PD, Zauber AG, et al.. Risk of ovarian cancer according to use of antidepressants, phenothiazines, and benzodiazepines (United states). Cancer Causes Control (2000) 11(9):839–45.
